# The Pathways to Healthy Ageing: Evidence From Longitudinal Studies and Real-Time Data

**DOI:** 10.1093/geroni/igab046.1806

**Published:** 2021-12-17

**Authors:** Terry Y S Lum

## Abstract

The WHO has replaced its active ageing policy framework developed in 2002 with the new Healthy Ageing framework developed in 2015 and declared the decade between 2020 and 2030 as the Decade of Healthy Ageing. Healthy Ageing framework emphasizes the pivotal role of functional ability (FA) among older adults and conceptualizes that FA can be determined by intrinsic capacity (IC), environments (EN), and their interaction. WHO calls for global research to advance theoretical understanding of Healthy Ageing framework and translate the evidence into policy actions. This symposium provides the latest findings on Healthy Ageing from multi-country studies using real-time data and longitudinal study design. Dr. Röcke explored daily time-out-of-home and place visit diversity with daily emotional and stress processes in Zurich, using sensor-based and self-reported mobility and activity indicators to capture FA. Dr. Lu investigated the EN and 4-year trajectories of IC and their impact on FA trajectories among older adults in Hong Kong. Dr. Liu explored the longitudinal associations between neighborhood physical EN and depressive symptoms of older adults in Hong Kong and the moderating effects of terrain slope and declining daily activity of living. Dr. Guo investigated the relationship between perceived EN (environmental cognition) and mental health and the mediating roles of physical activity and place attachment. Dr. Chan explored neighborhood physical EN and cognition among older people and identified whether this association varies among different older age groups. Based on these findings, this symposium will discuss the future research direction on Healthy Ageing and its policy implication.

